# P02.78. Treating pediatric asthma with holistic approaches of traditional Chinese medicine

**DOI:** 10.1186/1472-6882-12-S1-P134

**Published:** 2012-06-12

**Authors:** L Lo, S Chang, C Lee, T Lee, T Cheng, M Sun

**Affiliations:** 1 Changhua Christian Hospital, Department of Chinese Medicine, Changhua City, Taiwan; 2 National Changhua University of Education, Changhua City, Taiwan; 3 China Medical University Hospital, Taichung, Taiwan

## Purpose

Asthma is a chronic disease increasingly found in children. In order to find more economical and efficient alternatives to treat pediatric asthma, the Bureau of National Health Insurance of Taiwan launched the Traditional Chinese Medicine Holistic Treatment Program (TCMHTP). Based on the data collected during the program, we evaluated the effect of TCM holistic treatments on pediatric asthma.

## Methods

We performed a retrospective study by analyzing a dataset from Changhua Christian Hospital in Taiwan during January 1, 2006 and December 1, 2010. Patients aged between 2 and 15 years old who were diagnosed with asthma and have participated in TCMHTP were recruited, while those with other severe diseases were excluded. We analyzed the frequency of emergency department visits (EDV), inpatient admission rate (IAR), and length of hospitalization (LH) of the patients before and after TCM treatments. We also carried out spectral analysis of heart rate variability (HRV).

## Results

Fifty-eight patients were recruited. The average age of the patients receiving TCM treatments is 5.67±3.03 years old. The frequency of EDV reduced from 0.97±0.85 to 0.69±1.22 times annually (p=0.095), the IAR decreased from 0.86±0.81 to 0.36±0.77 times annually (p=0.001) and the average LH reduced from 4.59±4.43 to 1.10±1.86 (p=0.000) days per year. Parasympathetically mediated HRV reduced significantly from 60.42±15.33 to 54.89±16.45nu (p=0.016).

## Conclusion

In the present study, we found that an appropriate period of TCM holistic treatment intervention can not only significantly lower exacerbations and hospitalization frequency but also decrease vagal tone of the asthmatic children.

